# Surgical Opportunism for Hand Reconstruction Following a Mutilating Injury: A Case Report

**DOI:** 10.7759/cureus.63859

**Published:** 2024-07-04

**Authors:** Krishna A Patel, Tuna Özyurekoglu

**Affiliations:** 1 Surgery, OhioHealth Riverside Methodist Hospital, Columbus, USA; 2 Hand Surgery, University of Louisville, Louisville, USA

**Keywords:** spare parts, fillet finger flap, pedicled finger transfer, metacarpophalangeal joint reconstruction, free joint graft, non-vascularized joint transfer

## Abstract

We report our management of a 53-year-old female who suffered a wood planer hand-mutilating injury with significant dorsal soft tissue loss and partial metacarpophalangeal joint (MCPJ) amputations of the thumb, index, and middle fingers. The middle finger was deconstructed for “spare parts” and a vascularized osteochondral graft was utilized to reconstruct the metacarpal articular surface of the index finger proximal phalanx, allowing the pedicled transposition of the index finger to the third metacarpal. The middle finger's distal interphalangeal joint was transplanted non-vascularly to recreate the thumb MCPJ and the elevation of a middle finger fillet flap allowed dorsal wound coverage. The patient did well initially but required ulnar collateral ligament reconstruction with a palmaris longus tendon graft following MCPJ instability 10 months postoperatively. Nonetheless, she progressively regained thumb opposition and pinch grip and continues to have successful aesthetic and functional outcomes six years postoperatively, supporting the efficacy of non-vascularized joint transfers when vascularized options are superfluous or unavailable.

## Introduction

The aesthetic and functional restoration following a mutilating hand injury is a complex yet rewarding reconstructive task. Comprehensive anatomical knowledge, respect of anatomical variations, and sound technical skills allow for the innovative yet successful repurposing of disfigured skin, bone, tendons, and neurovasculature. The surgical management of a traumatic hand joint injury ranges from amputation, arthrodesis, arthroplasty, and joint transfer [1,2].

Joint transfers are further characterized by source (allogenic or autologous), origin (homodigit, heterodigit, or metatarsophalangeal), vascularity type (pedicled, free-flap anastomosis, or non-vascular), and amount of joint replaced (whole or partial). The reconstructive method utilized depends on factors involving both the trauma (mechanism, deformity, overall clinical stability) and patient demographic (age, profession, comorbidities, and motivation) [3].

Although vascularized joint transfers are preferred to reduce long-term degeneration, they may be deemed technically inappropriate based on the above factors or tissue availability. In this case report, we present the successful utilization of the middle finger to provide “spare parts” for the reconstruction of the thumb and index metacarpophalangeal joints (MCPJ) via a non-vascularized joint transfer and vascularized pedicle-based parts with satisfactory aesthetic and functional outcomes six years after injury.

## Case presentation

**Figure 1:**
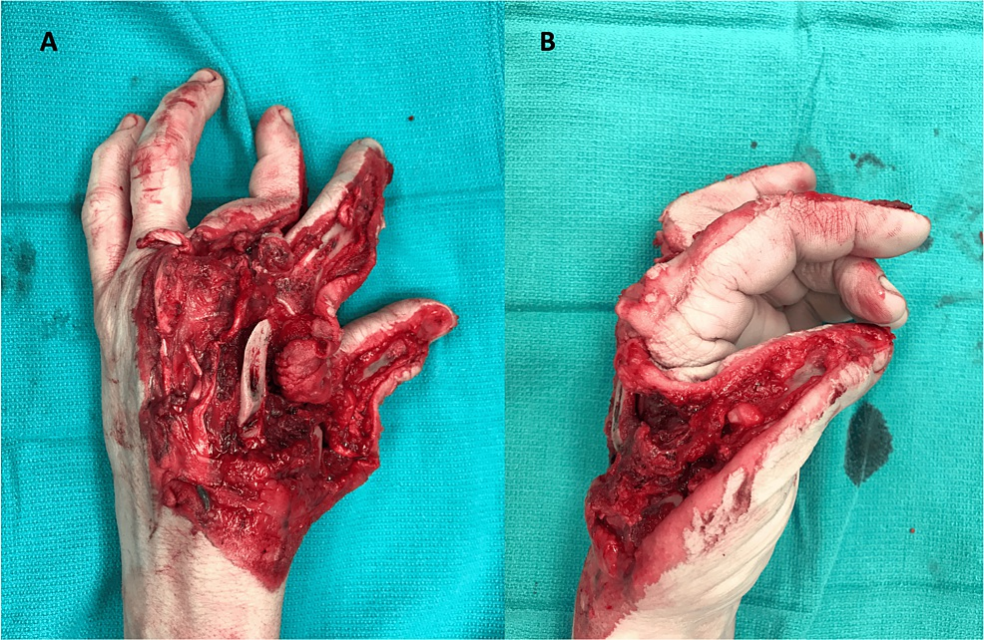
Traumatic hand injury resulting in significant loss of dorsal soft tissue and amputations of the metacarpophalangeal joints of the index and middle fingers (A) as well as the thumb (B). Image Credit: Tuna Özyurekoglu

A 53-year-old non-diabetic, non-smoking, right-handed female mutilated her left hand while operating an industrial wood planer and presented with a large dorsal soft tissue and bony defect with partially amputated thumb, index, and middle fingers (Figure 1). The patient underwent an axillary block, followed by tourniquet placement, and wound irrigation and debridement in the operating room. 

**Figure 2:**
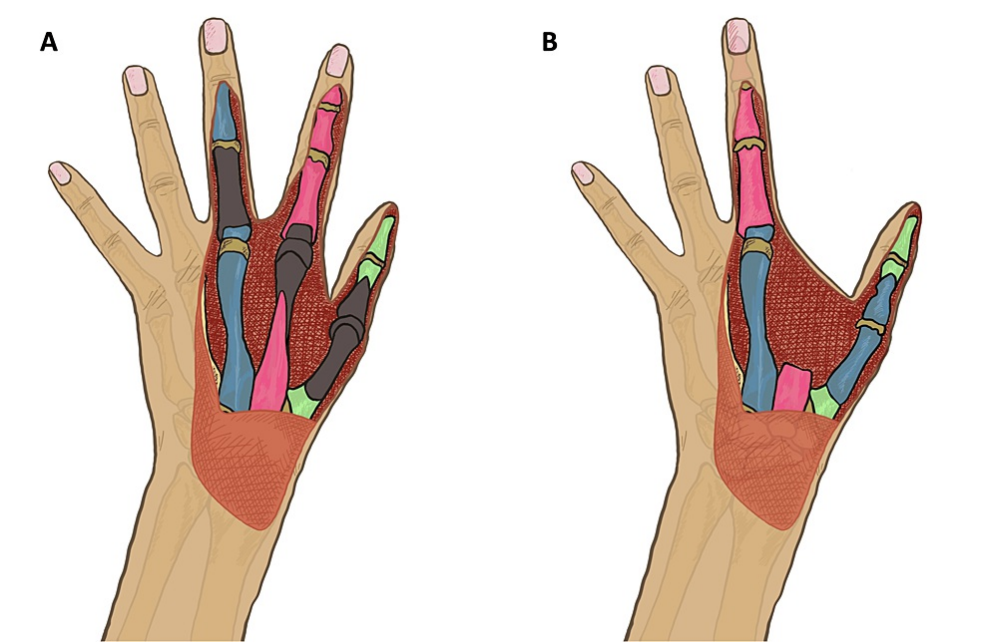
Traumatic loss (gray) of the thumb (green), index finger (pink), and middle finger (blue) are seen in panel A with their ultimate reconstruction demonstrated in panel B. Image credits: Krishna Patel

In the thumb, the skin, extensors pollicis longus and brevis, the distal half of first metacarpal and MCPJ were missing, such that only the volar proximal phalanx, the interphalangeal joint, and distal phalanx remained. The index finger MCPJ was mostly destroyed following amputation of the dorsal skin, extensor tendons, distal second metacarpal, and 75% of the dorsal articular surface of the proximal phalanx; however, the distal proximal, middle, and distal phalanges were uninjured. The middle finger lacked the majority of the proximal phalanx with its skin and extensor tendons, but a small osteochondral remnant remained at the MCPJ and the middle and distal phalanx were unharmed. The middle finger radial digital nerve and artery were disrupted. The traumatic defects and their ultimate reconstruction are respectively demonstrated in Figure 2.

**Figure 3:**
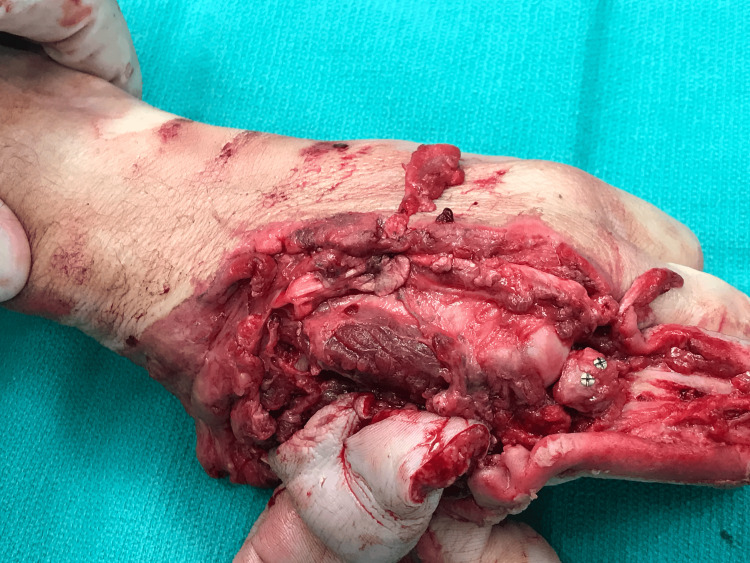
An osteochondral fragment of the middle finger metacarpophalangeal joint was secured to the proximal phalanx of the index finger with two 1.7 mm screws allowing for restoration of its articular surface. Image Credit: Tuna Özyurekoglu

To repair the index finger, the osteochondral MCPJ fragment from the middle finger was excised and secured onto the index proximal phalanx via two 1.7 mm screws, restoring its articular surface (Figure 3). The index extensor mechanism defect between zones III-V was restored with a tendinous graft from the middle finger, and the reconstructed index finger was transposed on its pedicle to the third metacarpal. The second metacarpal was truncated proximally. Following the repair of the joint capsule and the radial/ulnar collateral ligaments, a stable MCPJ was achieved. A radial and ulnar flap (8 x 2 cm) was mobilized and transposed to cover the index finger dorsal skin defect.

**Figure 4:**
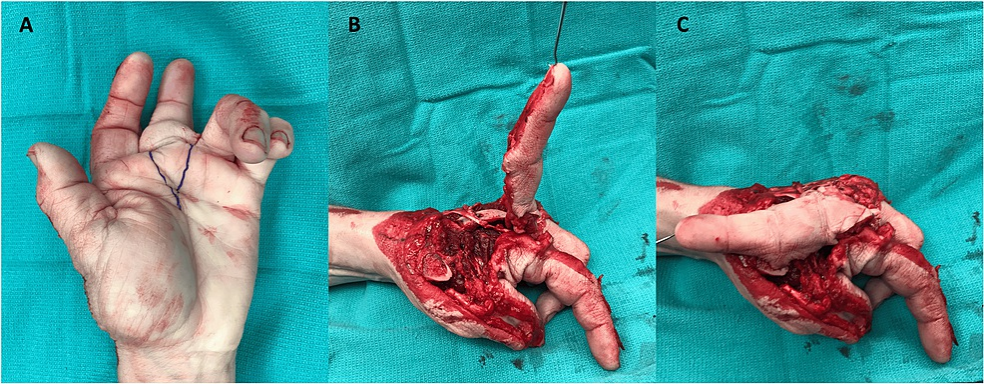
A V-shaped skin incision on the volar base of the middle finger was made (A) and a fillet flap was raised on the ulnar digital artery and nerve (B), which later allowed for dorsal soft tissue wound coverage (C) Image Credit: Tuna Özyurekoglu

**Figure 5:**
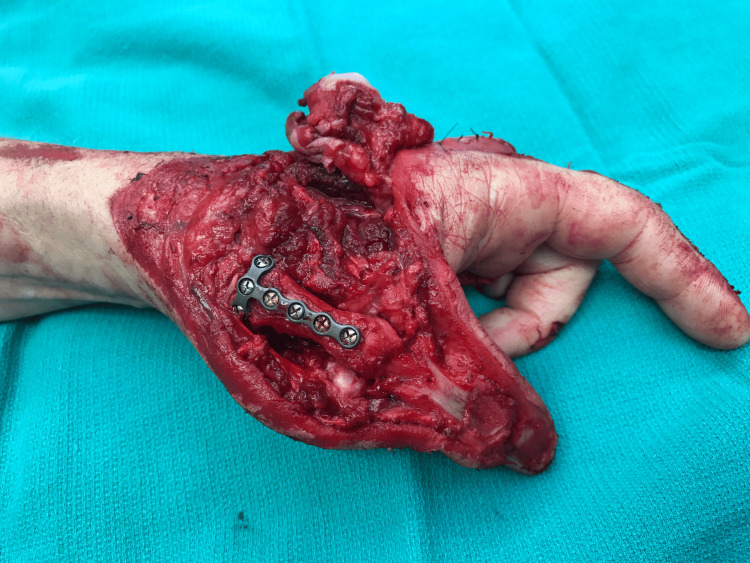
The proximal articular surface of the middle phalanx was transfixed to the base of the first metacarpal with a 2.3 mm T-plate with locking screws proximally and locking and compression screws distally. Image Credit: Tuna Özyurekoglu

**Figure 6:**
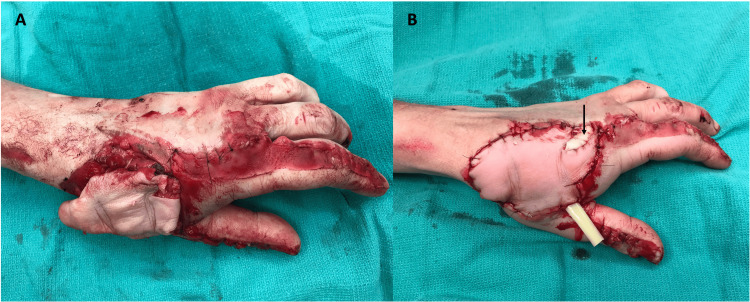
Dorsal wound coverage obtained through the combination of a fillet graft (A) and a small full-thickness skin graft obtained from the fillet graft (B, arrow). Image Credit: Tuna Özyurekoglu

A V-shaped skin incision on the volar base of the middle finger and a fillet flap (8 x 6 cm) was raised on the ulnar digital artery and nerve, which would later be used for wound coverage (Figure 4). For thumb MCPJ reconstruction, we sharply transected the flexor tendons of the middle finger and excised the distal interphalangeal (DIP) joint along with the intact middle and distal phalanx for a free joint transfer. The proximal phalanx of the thumb was already lacking cortical bone dorsally, and after drilling a hole in the medullary canal, the middle finger distal phalanx was inserted into the thumb proximal phalanx and secured with a 1.7 mm screw. The proximal articular surface of the middle phalanx was removed with an oscillating saw and transfixed to the base of the first metacarpal with a 2.3 mm T-plate with locking screws proximally and locking and compression screws distally (Figure 5). The extensor pollicis brevis was transferred to the extensor pollicis longus and transfixed onto the distal phalanx of the thumb via a micro-corkscrew. A nailbed graft (0.8 x 1 cm) from the middle finger was secured on the radial aspect of the thumb with a chromic gut suture. The dorsal skin defect on the thumb was closed by the transposition of two 5 x 1.5 cm radial and ulnar flaps. The previously mentioned middle finger fillet flap maintained satisfactory circulation and was utilized for dorsal radial wound coverage. A small portion (2 x 1.5 cm) of the fillet graft was excised and applied as a full-thickness skin graft to cover the remainder of the defect (Figure 6). A sterile dressing and a volar thumb spica splint were applied and the patient was admitted for flap observation and antibiotics.

**Figure 7:**
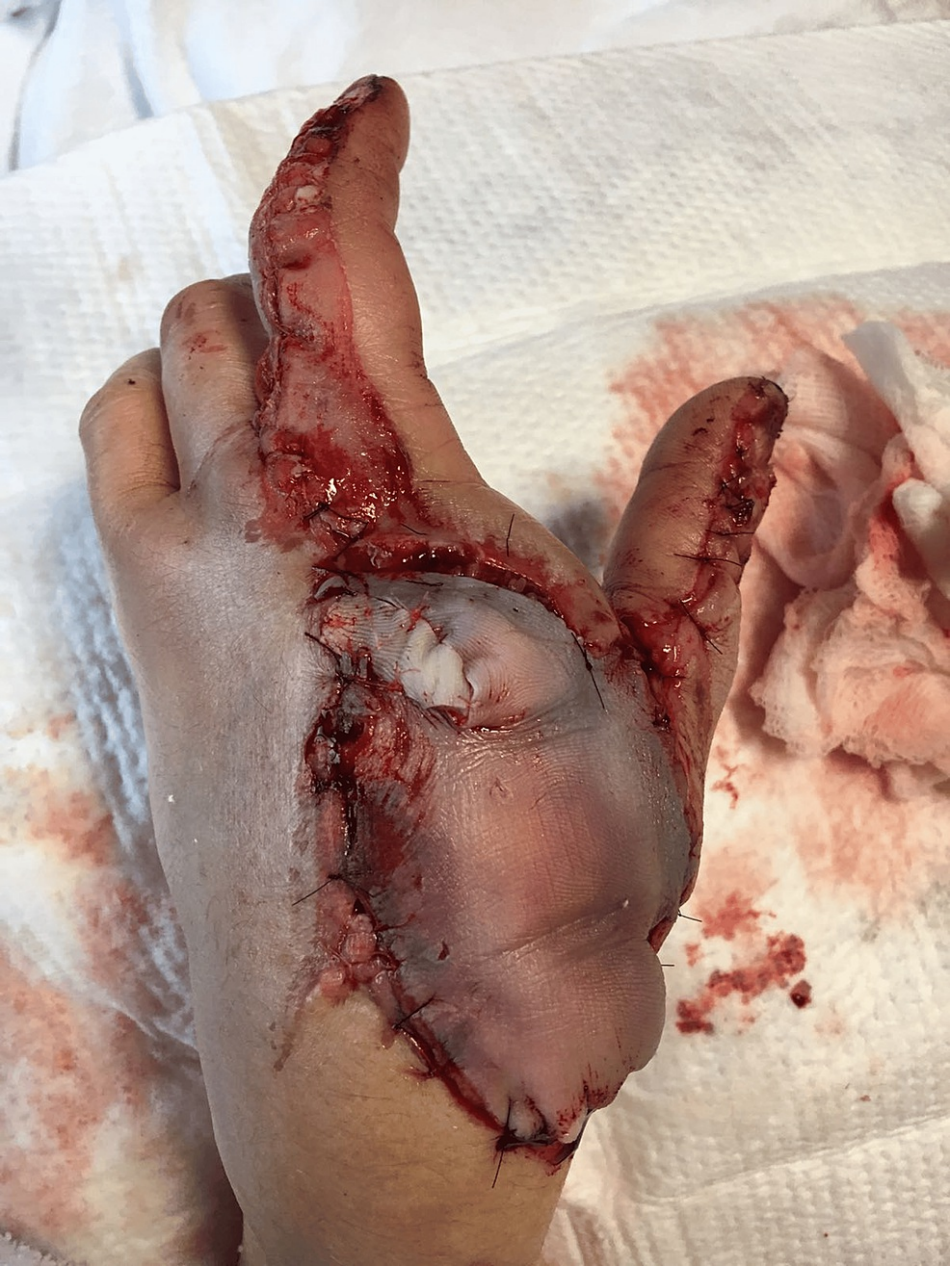
Mild venous congestion and sloughing of the fillet flap seen postoperatively which ultimately underwent leech therapy. Image Credit: Tuna Özyurekoglu

Postoperatively, mild venous congestion and sloughing of the fillet flap occurred as expected and leech therapy was initiated (Figure 7). She was discharged on postoperative day 6 on levofloxacin (750 mg once a day) and cefalexin (500 mg four times a day) for *Aeromonas* infection. She progressively regained hand dexterity through extensive postoperative physical therapy. Unfortunately, ulnar collateral ligament (UCL) instability at the thumb and contracture of the first web space was noted at 10 months postoperatively and she underwent UCL reconstruction with a palmaris longus tendon graft and a double Z-plasty with adductor myotomy.

**Figure 8:**
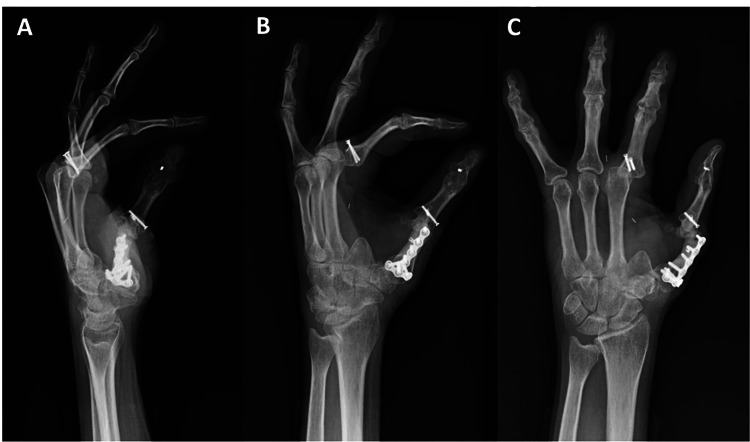
Mulitiple radiographic views (A-C) demonstrating the patient's index finger subluxation at clinic follow-up. Image Credit: Tuna Özyurekoglu

**Figure 9:**
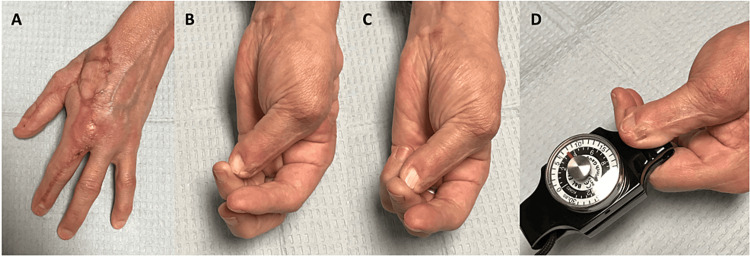
At the two-year postoperative follow-up, the patient demonstrated satisfactory postoperative aesthetic outcomes of the dorsal hand (A) as well as intact opposition to the small finger (B), ring finger (C), and pinch grip (D). Image Credit: Tuna Özyurekoglu

By her two-year follow-up visit, she demonstrated satisfactory aesthetic and functional outcomes, with good thumb opposition to all remaining digits and intact pinch strength. Although index finger MCPJ subluxation was noted on X-ray (Figure 8), the patient maintained a strong pinch grasp and thumb opposition to all digits (Figure 9). She continues to be functionally and aesthetically satisfied with her outcome six years following her injury. 

## Discussion

As seen in our patient, the finger contains the same fundamental tissues as the rest of the hand and is a valuable source of spare tissue. After identifying the damaged radial digital artery and nerve of the middle finger, we decided to deconstruct and repurpose the middle finger to fix the defects involving the thumb and index finger. The osteochondral remnant of the middle finger proximal phalanx allowed for the reconstruction of the index finger proximal phalanx metacarpal articular surface. Extensor tendons from the middle finger helped graft the index finger extensor mechanism. The distal phalanges and DIP joint of the middle finger allowed for thumb MCPJ reconstruction. The nailbed from the middle finger was transplanted to the thumb and the skin fillet flap raised on the ulnar digital artery and nerve provided sensate soft tissue coverage of the dorsal hand. 

The MCPJ contributes significantly to hand dexterity, finger flexion, and thumb opposition for pinch grip and is an operative challenge to reconstruct following traumatic destruction. Surgical management ranges from amputation, arthrodesis, arthroplasty, or joint transfer [1,2]. MCPJ or interphalangeal joint grafts can be further divided into synthetic, allogeneic, or autologous grafts (which include vascularized or non-vascularized joints). Vascularized joint grafts can be raised on a pedicle or can be a free graft which is anastomosed microsurgically from the same finger (homodigital), a different finger (heterodigital), or the patient’s toes. Both non-vascularized and vascularized grafts can provide a whole joint (complete) or hemi-joint (partial) replacement.

If the finger is salvageable, arthrodesis of the carpometacarpal, thumb MCPJ, or long finger interphalangeal joint can provide a pain-free stable joint at the cost of mobility [1]. Implant arthroplasty may be a good option in some patients but is associated with high complication rates, precluding widespread utilization in acute trauma [4]. Similarly, allogenic joint transplants have demonstrated favorable outcomes in animal models but have yet to be reliably studied in humans and clinical use is limited by the necessity of lifelong systemic immunosuppression [5]. Vascularized MCPJ transfers can be harvested from spare non-replantable fingers (homodigital or heterodigital) or the metatarsophalangeal joint (MTPJ) and includes concurrent transplantation of the associated ligaments, bones, capsule, and overlying skin with satisfactory outcomes [6,7]. Pedicle-based vascular joint transplants avoid the time and technical burden of microsurgical anastomosis compared to free joint transplants but still require meticulous dissection and may not always be anatomically available [5,8]. Long-term outcomes are better in complete joint compared to hemi-joint transplants due to better cartilage preservation, synovial safeguarding, and avoidance of incongruent joint surfaces and are preferred if obtainable [5].

In our patient, utilization of the middle finger metacarpophalangeal osteochondral remnant and the DIP joint were used to reconstruct the MCPJ of both the thumb and index, respectively. Though non-vascularized joint transfers carry a higher risk of deterioration and avascular necrosis, they can be an effective reconstructive option with good outcomes and patients may remain asymptomatic despite radiologic evidence of degeneration [5,8]. In the present case, the patient continues to demonstrate satisfactory outcomes six years following her injury.

## Conclusions

Complex mutilated hand injuries present a formidable challenge for reconstructive surgeons as they are tasked with restoring both aesthetic form and function amongst remnants of disfigured tissue. Unsurprisingly, comprehensive anatomical knowledge, meticulous technique, and utilization of the hierarchal reconstructive ladder are essential for favorable recovery and rehabilitation post injury. This case illustrates the simplicity and effectiveness of non-vascularized joint transfers and supports their use when vascularized reconstructive options are unavailable, superfluous, or confer unacceptable donor site morbidity.
